# Research Advances on Hepatotoxicity of Herbal Medicines in China

**DOI:** 10.1155/2016/7150391

**Published:** 2016-12-18

**Authors:** Changxiao Liu, Huirong Fan, Yazhuo Li, Xiaohe Xiao

**Affiliations:** ^1^Research Center for New Drug Evaluation, Tianjin Institute of Pharmaceutical Research, Tianjin 300193, China; ^2^Institute of Radiation Medicine, Chinese Academy of Medical Sciences & Peking Union Medical College, Tianjin 300192, China; ^3^China Military Institute of Chinese Medicine, Integrative Medical Center, 302 Military Hospital of China, Beijing 10003, China

## Abstract

In general, herbal medicines have been considered as safe by the general public, since they are naturally occurring and have been applied in treatment for over thousands of years. As the use of herbal medicine is rapidly increasing globally, the potential toxicity of herbal drugs, in particular drug-induced liver injury (DILI), has now become a serious medical issue. According to the literature, the authors analyzed and discussed the hepatotoxicity problem of Chinese herbal medicines (CHM), including global overview on herbal-induced liver injury (HILI), current research progress on toxic CHM, diagnosis and treatment of HILI, and modern approaches and technologies of study of hepatotoxicity. As to promote the recognition of HILI and tackle the issue, a guideline for the diagnosis and treatment of HILI has recently been drafted by Chinese scientists. As suggested by the guideline, the hepatotoxicity issue of CHM, as a matter of fact, is overestimated. Up to date, the investigation of hepatotoxicity of CHM is now booming with worldwide application of CHM. This review therefore provides useful information for investigating hepatotoxicity of herbal medicine and characterizing DILI caused by CHM. In addition, authors describe in which way further efforts should be made to study the rationale of CHM and liver injury.

## 1. Introduction

In general, herbal medicines have been considered as safe by the general public, since they are naturally occurring and have been applied in treatment for over thousands of years. As the use of herbal medicine is rapidly increasing globally, the potential toxicity of herbal drugs, in particular drug-induced liver injury (DILI), has now become a serious medical issue. Many herbal medicines exhibit pharmacological activities and toxic effects by chemical substances as secondary metabolites. Some herbal medicines have been associated with hepatotoxicity, which is reversible upon discontinuation of the treatment by patients [1].

A large number of studies have identified chemical substances with a hepatotoxic effect, which has been linked to liver injury; therefore, the hepatotoxic effect is defined as drug-induced liver injury (DILI). Oh et al. analyzed herbal-induced liver injury (HILI) by performing literature review from eight databases, including PubMed, Medline, the Cochrane Library, EMBASE, and four Korean electronic databases. Based on the comprehensive data, the analysis indicated the incidence of hepatotoxicity in patients using herbal drugs and the possibility of increased risk of HILI by coadministration of herbal and conventional medicines [2]. In China, herbal hepatotoxicity and diagnosis of HILI have been reported widely from preclinical studies to clinical observation. However, HILI study remains still rather difficult.

In this paper, the authors provide an analysis and discussion of the hepatotoxicity of CHM. They also include a global overview of herbal-induced liver injury (HILI), current research progress on toxic CHM, diagnosis and treatment of HILI, and modern approaches and technologies used to study hepatotoxicity. The investigation of CHM hepatotoxicity is now booming with worldwide application of CHM; however, the hepatotoxicity of CHM is overestimated. A guideline for the diagnosis and treatment of HILI has now been drafted. This review therefore provides useful information for investigating hepatotoxicity of herbal medicine and characterizing DILI caused by CHM and outlines the direction of further efforts in the study of CHM and liver injury.

## 2. Research Overview on DILI/HILI

Over 30 herbal medicines were reported to cause DILI by LiverTox, a database maintained by the US National Library of Medicine (Table 1). Meanwhile, herb-related products were ranked as the second among the most common causes to cause DILI in the US based on the recent guideline published by the American College of Gastroenterology (ACG) [3].

However, in clinical practice, the definitive DILI diagnosis is extremely difficult because there are not a test and criteria existing for accurate diagnosis. RUCAM (Roussel Uclaf Causality Assessment Method) [4], along with the Maria and Victorino scale [5], and the structured methodologies based on expert opinion proposed by the US DILIN (Drug-Induced Liver Injury Network) recently, are the most popularly used methods for assessing the causality between liver injury and the implicated medications [6]. Unfortunately, test-retest reliability and interrater reliability were reported as 0.54 and 0.45, respectively, when using the RUCAM scales reported by a study from the DILIN, suggesting that the reliabilities of applying such standardized causality assessment methods are rather low [7].

Although identifying all medications is a vital element in determining DILI causality, it is extremely difficult to achieve for an accurate DILI assessment if solely relying on patient recall [8] because of increased polypharmacy from multiple independent providers. As for herbal DILI, this scenario is particularly complicated due to polypharmacy and the combined use of chemical pharmaceuticals.

As reported by WHO (World Health Organization) [9], herbs in the marketplace are frequently contaminated with the hazardous materials (e.g., heavy metals, mycotoxins, and pesticides), alternative plant species, and fillers that are not listed on their labels, which could cause misidentification of causality and lead to confounding of the scientific diagnosis of herbal DILI, overdiagnosis, and overreporting [10]. For example, the hepatotoxicity of black cohosh [11, 12] and* Pelargonium sidoides* [13] have been suspected with controversial arguments from the confounding variables. As commented by Dr. Teschke et al. [14], quality is far more important than the quantity for the causality assessment. So far, the universal causality assessment methods (e.g., the RUCAM scale) still heavily rely on the exclusion of other causes of liver injury, and consequently the identification of the real risk associated with herbal DILI has been minimally addressed.

## 3. Long-Term Application of Toxic Drug in China and Current Status of Toxic CHM Research

### 3.1. Documented Toxic CHM in China

“*Zhou day official*” (*Zhouli Tiangong *in Chinese), an ancient medical book, recorded that physicians are in charge of the government and apply poly poisons for medical use, which reflects the concept of coexistence of therapeutic effects and adverse reaction of drugs.* People's Republic of China Pharmacopoeia *(2005 Edition) contains 72 toxic drugs, including 10 toxic medicines, 38 moderate toxic medicines, and 24 mild toxic medicines.  Table 2 lists the typical toxic Chinese herbal medicines originated from including medicinal herbal plants, minerals, and animals.

### 3.2. Chinese Medicines with HILI in Animal Experiments

Chinese medicines with HILI that have been identified in animal experiments are listed in Table 3.

In addition, some topical medications, such as fish guts, rotenone, and realgar, have been identified with liver toxicity in animal studies which may lead to liver damage with varying degrees if taken orally. Since the toxic dose is far more over the amount used in the treatment, the possibility of occurrence of toxicity remains at very low level. Apart from this, the hepatotoxicity of certain herbal medicines cannot be confirmed, due to lack of reliable clinical evidence.

### 3.3. Chinese Medicines with HILI in Clinics

Chinese medicines associated with HILI in clinical use have been reported. Chinese medicines associated with HILI in clinical use have been reported as shown in Table 4. There are also several known CHM compound formulations associated with liver injury in clinical observation. CHM formulations with DILI are listed in Table 5.

For example,* Polygonum multiflorum *Thunb. (*Heshouwu* in Chinese), which is officially listed in the Chinese Pharmacopoeia, is one of the most popular perennial Chinese traditional medicines but is also associated with HILI. Currently, pharmacological studies have unveiled its key benefits in the treatment of various diseases, providing information relevant to pharmacokinetics-pharmacodynamics analysis, neurodegenerative diseases, dyslipidemia treatment, and sleep disorders [18].

### 3.4. Categories of Chemical Substances with HILI

Herbal drug-induced liver injury (HILI) is now a hot spot in the field of CHM safety study. Rapid screening and evaluation of herbal drug-induced liver injury have become one of the key techniques of herbal research. In this section, the research status of HILI from active, toxic ingredient, mechanism of toxicity, and toxicity reduction of Chinese herbal drugs is discussed. According to the structural information of the chemical composition, toxic substances can be divided into the seven categories (in Table 6) [19].

### 3.5. Current Status of HILI in China

*Heshouwu* (dried root of* P. multiflorum*) has been traditionally used in China as a tonic for liver and kidney conditioning for thousands of years without significant adverse effects found. Currently,* Heshouwu* has been widely used to prevent hair loss and graying, prevent aging, and extend lifespan, which also has potential for treatment of Parkinson's disease, liver injury, hyperlipidemia, and Alzheimer's disease. However, a typical example of DILI causality determination of* Heshouwu* was presented by Wang et al. [15]. The major active ingredient might be 2,3,5,4′-tetrahydroxy trans-stilbene-2-O-*β*-glucoside (TSG), and it was found to expand the lifespan of model organisms by activating Sirtuin 1 (Sirt1) [20, 21] and cause autophagy stimulation [22]. In Hong Kong,* Heshouwu*-related DILI cases were identified in 1996 and consequently led to the announcement by the Medicines and Healthcare Products Regulatory Agency in 2006. Therefore, the reports related to* Heshouwu *DILI have caused increased concerns regarding the safety of herbs usage.

Currently,* Heshouwu* remained to be controversial in terms of its safety and effects, and functions and role of* Heshouwu* are not entirely conclusive, due to absence of excluding confounding DILI causality factors (such as heavy metals, pesticides, and fungal toxin contamination) and lack of pharmacognostic identification of patients' digested materials.

**Figure 1:**
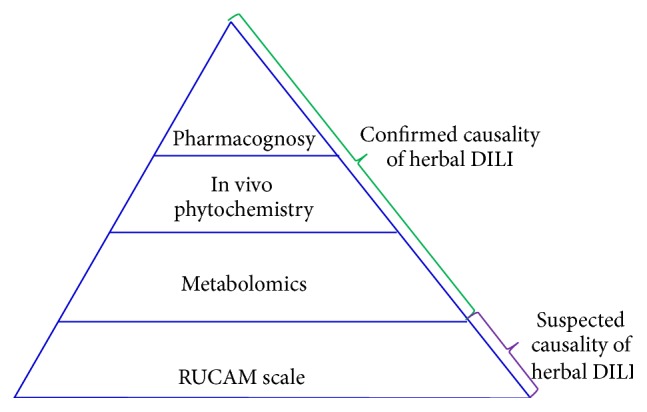
Proposed RUCAM-based stepwise strategy to causality assessment of herbal DILI [15].

Based on the general RUCAM assessment, Wang et al. established a model for assessing HILI causality that combines translational laboratory tests with pharmacognosy, phytochemistry, and metabolomics [15, 20–23] (Figure 1). This model might improve our capability for the diagnosis and causality assessment of HILI.

### 3.6. Safety Regulation of Chinese Herbal Products

For safe application of Chinese herbal products, apart from conventional investigation of mechanisms of safety, efficacy, and toxicity, regulation of Chinese medicine products is needed to enhance the recognition of safety in the following aspects: (1) understanding the role of Chinese herbal medicine treatment and adverse drug reactions (ADR) coexisting; (2) the proper use of CHMs; and (3) avoiding overdosing of herbs and unnecessary prolonged treatment, so as to reduce the incidence of ADR. Most importantly, active academic exchanges and cooperation with foreign regulatory agency, with respect to safety issues of herbal medicines, are urgently needed to promote communal understanding and communication. Safe and effective herbal medicines can then be recognized globally.

According to Chinese Drugs Act and ADR monitoring and reporting system, the appropriate research and monitoring medicine safety management system is established. The main work was done in the following six areas: (1) adverse drug reactions monitoring by strengthening communications to promote understanding of drug manufacturers, medical practitioners, and public; (2) introducing investigation methods of Chinese herbal medicine to improve the clinical application; (3) encouraging scientific groups to actively participate in society of herbal medicines, in terms of safety issues; (4) encouraging pharmaceutical companies to carry out postmarketing product safety reevaluation to enhance the effectiveness and safety of Chinese medicine; (5) performing hospital-based medicine safety monitoring; and (6) establishing the national and local research institutions to perform systematical studies on the clinical manifestations, mechanisms, and prevention measures of ADR of CHMs.

## 4. Diagnosis and Treatment of HILI in China

### 4.1. Understanding of Herbal-Induced Liver Injury

In recent years, with the rising worldwide use of herbs and the improvement of adverse drug reaction monitoring system, HILI reports rapid increase [3]. Factors affecting HILI are complicated and diverse. In addition, there are other factors, for instance, individual variation and irrational drug use [24]. Due to the lack of standards reflecting the complexity of HILI diagnosis, the current clinical diagnosis of HILI is often inaccurate. In addition, an integrated drug classification system has not been established for comparison of liver damage, resulting in a higher level of HILI [25]. Therefore, the establishment of guidelines for diagnosis of HILI and treatment with the characteristics of Chinese herbal medicine becomes very important for patients with hepatic impairment. Enhanced recognition of scientific objectivity and judgment of HILI may improve diagnosis and treatment, as well as reduction of liver damage, in particular for development of pharmaceutical industry.

### 4.2. Proportion of HILI, in Terms of DILI, Varies in Different Countries and Regions

These documents are associated with more than a single-center retrospective investigation and diagnosis center, which is related to different levels of HILI. In addition, the drug and level of liver damage caused are determined by the statistical methods adopted [26–33]. Most herbs are used as a whole or a certain type of pharmaceutical (such as anti-TB drugs) and are even compared to certain pharmaceuticals (such as acetaminophen), in terms of efficacy and safety. However, other ingredients of the herbs are not taken into account, leading to higher proportions of one-sided conclusions [34].

### 4.3. HILI Factors Affecting the Complexity and Diversity

Herbal factors affecting HILI complexity and diversity include improper clinical use, individual variation, and combination of pharmaceuticals.

*(1) Herbal Factors*. These factors include the following: (a) some herbs may produce direct damage on the liver, such as* Gynura japonica* L. (*Zusanqi *in Chinese) [33] and* Tripterygium wilfordii* Hook. f. (*Leigongteng* in Chinese) [35]; (b) variety mix: some herbs homonym, pseudo product mix, such as clinical application of* Gynura japonica *L. as* Panax notoginseng* can cause liver injury [36]; and (c) improper processing: unreasonable concocted herbal medicine may increase the risk of liver damage, such as nonstandard cooked or raw* Radix Polygonum. *Risk of liver injury is higher than that of the standard stipulated* Polygonum* [37]. Exogenous harmful substances: herbal drugs may be contaminated during growth, processing, storage, transportation, and other courses, resulting in seriously high level of herbal pesticide residues, heavy metals, and microbial toxins, which causes liver damage [38, 39]. Meanwhile, inadequate processing could augment the toxic risk of the clinical usage of* Radix Polygonum* [15]. 

*(2) The Irrational Clinical Use*. Chinese herbal medicine should be applied in accordance with the theory of CHM, according to the diagnosis and judgment made. Cross-checks of medication, dose selection, and appropriate compatibility of toxic drugs are necessary for treatment of diseases. Overdosed drug, unconventional dosing or treatment, and improper drug compatibility may increase risk of liver damage [40]. For example, not as other well-known hepatotoxic herbs, the potential risk of* Heshouwu* was not fully clarified in the clinical community and therefore the hepatotoxic risk among herbs should be paid attention to in clinical practice and by regulatory administration [15]. Patients with different physical conditions, for instance, underlying chronic diseases, genetic differences, and other factors, may also have increased risk of liver damage [41]. 

*(3) Combination with Pharmaceuticals*. Combination of herbal medicines and Western medicines may result in higher risk of liver damage. In treatment, it can lead to liver damage while taking chemical drugs such as cholesterol-lowering statin drugs. Some of the herbal drug preparation is actually in the compound formulation, which contain a hepatotoxic pharmaceutical ingredient, for example acetaminophen. Consequently, the liver injury caused by the combination of Chinese and chemical medicines cannot be simply attributed to herbal and related agents.

### 4.4. Clinical Features of DILI/HILI

DILI has been reported to be widely caused by a variety of pharmaceuticals, herbs, and other toxic substances. In Western countries, 1.2%–6.6% of acute liver disease cases reported at tertiary referral centers were related to DILI, and it also accounts for 13% of acute liver failure [42, 43]. According to the *R*-value between ALT (serum alanine aminotransferase) and ALP (alkaline phosphatase), clinical DILI cases were classified into hepatocellular, cholestatic, and mixed types [44]. In Western countries the common causative agents and their clinical chemistry type of DILI have been documented [45, 46].

Hepatocellular injury was mostly frequently cited as the liver injury caused by herbs, dietary supplements, and folk remedies. The etiology of DILI in Western countries usually differs from those in Asian countries, particularly in China and Korea. In Western countries, DILI were mainly caused by the prescription of chemical drugs (e.g., analgesics, antibiotics, and CNS agents) [47, 48]. The ingredients of prescription drugs are generally known, while the exact causative ingredients among the herbs and folk remedies used in traditional medicine remain unclear [49]. Similar with prescription medications, the liver injury type observed among herbal and dietary supplements is predominantly hepatocellular too [50]. Thus, the *R*-values based methodologies for DILI classification might not be an appropriate approach for Asian patients as compared with the patients in Western countries.

**Figure 2:**
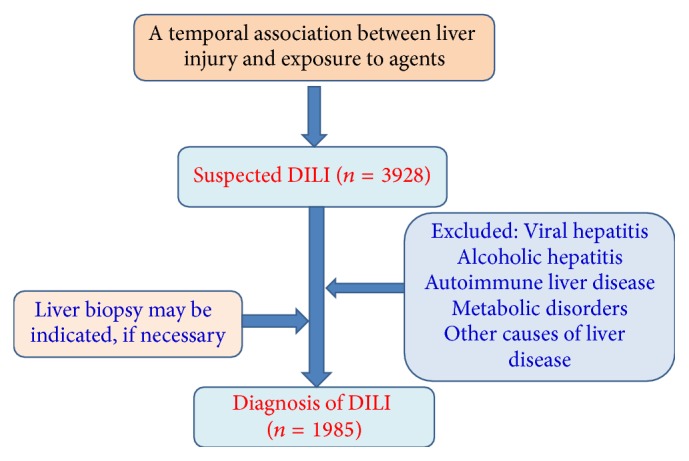
The flowchart illustrating drug-induced liver injury diagnosis.

In a case study of DILI reported by 302 military hospitals from 2009 to 2014, of 96857 inpatients, 1985 DILI cases with liver dysfunction were retrospectively collected. The portions of patients prescribed CHM, pharmaceuticals, and combination of both were 28.4, 43.8, and 27.8%, respectively. By comparison with pharmaceuticals, the DILI caused by CHM presented a higher mortality, but there was no significant difference, in terms of rates of chronic DILI and acute liver failure (12.9 versus 12.4%, *P* = 0.807; 7.6 versus 7.6%, *P* = 0.971). Additionally, 75.6% of cases caused by CHM were considered as probable. The highly probable cases were only 16.6%, based on Roussel Uclaf Causality Assessment Method. The diagnostic criteria used have been illustrated in Figure 2 [25].

### 4.5. Risk Factors and Future Challenges on HILI Research

**Figure 3:**
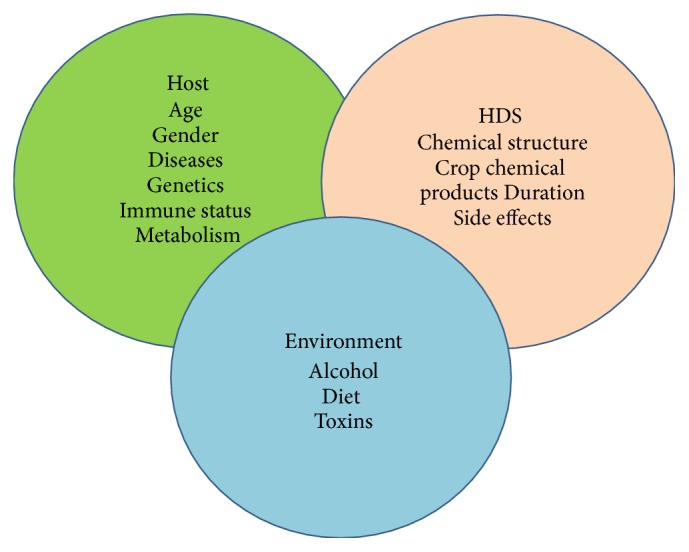
A variety of theories related to the pathogenesis of HILI [16].

A large portion of DILI reported in Western countries is HILI. Several risk factors for HILI, for instance, components of herb, their side effects, and pesticides used on the crop and other environmental factors should therefore be given special consideration. In pathogenic aspects of HILI, there are several theories about the pathophysiology of liver injury, the majority of which are based on DILI. The “damage hypothesis” suggests that HILI may be caused by the following aspects: (1) formation of parent drug-protein complexes, (2) irreversible generation of reactive metabolites, (3) inhibition of the bile salt export pump, and (4) intracellular damage indirectly mediated by oxidative endoplasmic reticulum stress and mitochondrial damage. The “hapten hypothesis” suggests that (1) metabolite-protein and (2) the drug-protein adducts lead to inadvertent activation of the adaptive immune system.  Figure 3 depicts a variety of theories related to the pathogenesis of HILI. The overlap is mostly with those for DILI in host, herb-drug supplements (HDS), and environment factors [16, 51].

Up to date, a large portion of medicines used in developing countries are herb drugs. It is unlikely to evaluate safety of herbal drugs. Herbal medicines are considered to be safe though liver injury may still be a potential risk. It is difficult to determine the exact incidence of HILI, which might have been seriously underestimated. As to determine the toxicity and adverse effects, multiple components are needed to be carefully separated, combined with ethnopharmacological and toxicological investigations. Therefore, the recognition of indicative symptoms of liver dysfunction is important because a potential differential diagnosis of the liver injury could improve the patient prognosis.

### 4.6. Chinese Guideline for Diagnosis and Treatment of HILI

At present, liver histopathology does not establish the diagnosis of DILI with the required certainty. To fulfill the requirement of diagnoses of DILI and HILI, clinical and structured causality assessments are the better approaches than liver histopathology results obtained from liver biopsy, an invasive procedure with a low complication rate. Scientists and physicians now face difficulties and challenge for HILI diagnosis. HILI is a hot issue in the field of CHM study. We thought that this guideline has a great significance since it could make clear the correlation of liver injury and CHM, improve the diagnosis and treatment level of HILI, guide the rational application of CHM, reduce occurrence of HILI, and promote healthy and sustainable development of CHM. For the past 10 years, the Chinese medicine experts have been dedicated to this research, making a significant contribution in the following two aspects. 

*(1) Rapid Screening and Evaluation of HILI Have Become One of the Key Techniques of Herbal Research*. For example, Li et al. analyzed the toxic ingredient, mechanism of toxicity, and reduction of toxicity of CHM [19]. The studies performed highlighted how to investigate toxicity of CHM with a scientific approach and rational. First, the toxic effects of CHM with HILI diminished or disappeared by reducing the level of toxic ingredients or by modifying the chemical structure. Second, through the research on the integrated evidence chain-based identification strategy for HILI and the CHM characteristic-oriented toxicity attenuating and rational use strategy, it would be helpful to provide scientific support for HILI diagnosis and CHM prescription and to promote integrated solutions for individualization therapy of clinical precision medicine using CHMs [52]. 

*(2) The Released “Guideline for Diagnosis and Treatment of Herbal-Induced Liver Injury.” *Since there are still many issues, in terms of HILI diagnosis and treatment, the China Association of Chinese Medicine (CACM) organized experts and developed “Guideline for diagnosis and treatment of herbal-induced liver injury,” which requires constant revision and improvement under the support of evidence-based medical science. This guideline describes HILI terms and definitions, epidemiological studies, affecting factors, clinical characteristics and type of injury, histopathologic features, the degree of damage classification, differential diagnosis, diagnosis strategies and methods, diagnostic criteria, and others. Currently, the guideline has been approved and published by CACM (T/CACM 005-2016) in April 2016 [53].

In this guideline, the Integrated Evidence Chain-Based Causality Identification Algorithm (IECCIA), a new perspective and workflow, was proposed and recommended in HILI diagnosis for Chinese medical practitioners, which are composed of five nodal segments: (1) thorough exclusion of confusing liver diseases; (2) thorough history review of implicated drugs, especially excluding the combination of Western medicines and CHM; (3) pharmacognostic identification and quality assessment for the implicated herbs, including plant origin, exogenous toxin contamination, and synthetic drugs adulteration; (4) detection of characteristic in vivo metabolites of implicated herbs; and (5) detection of specific HILI biomarkers of implicated herbs. Based on this workflow, the guideline proposed the three-level diagnosis system for the first time, suspected, clinical, and confirmed diagnosis for HILI. Some misunderstandings of HILI and the rational usages of CHM were also explained elaborately in the guideline. Notably, the proposed HILI diagnosis workflow could also be used in diagnosing DILI to obtain integrated evidence chain.

The proposed HILI guidelines with the characteristics of Chinese medicines for liver damage and clear relationship between medicine and scientific judgment of HILI are objective in terms of improving the diagnosis and treatment, clinical therapy, and modern scientific research. CACM experts suggest a scientific and rational classification of comparison of drugs with liver injury: a classification would divide drugs with induced liver injury into Chinese herbal medicine, small molecule pharmaceuticals, and biologics; a secondary classification could classify Chinese herbal medicine and pharmaceuticals, according to their comparative efficacy; a third tier would compare a particular species of herb to its pharmaceutical [53].

## 5. Modern Methods and Technologies of Hepatotoxicity Study

Accurately predicting the potential hepatotoxic characteristics of pharmaceutical products remains a challenge. Therefore, improved models, methods, and technologies are needed to study liver metabolism and identify drug hepatotoxicity in humans.

### 5.1. Drug Metabolism Enzymes and Liver Enzymes

Herbal medicines are a complex system. Due to the diversity and complexity of their chemical composition, the same chemical substances in various pharmaceutical formulations exhibited differences in drug metabolism and dynamic behavior [54–59]. Such differences are a reflection of drug-drug interactions (DDIs) or herb-drug interactions (HDIs). The complex results of DDIs or HDIs are related to transporters and metabolizing enzymes.

Cryopreserved human hepatocytes or AREHCs (Assay-Ready Expanded Hepatocytes) are widely applied in researches for hepatic metabolism, CYP induction/inhibition, compound uptake, genotoxicity, hepatotoxicity, and 3D coculture [60]. AREHCs have functional CYP activity. Therefore, AREHCs are qualified for study of CYP induction, metabolism [57]. AREHCs are comparable to HepaRG cells, in terms of basal phase I enzyme activities. AST, ALT, GLDH, and LDH are measured for the indication of liver injury, and these enzymes are released upon membrane leakage when hepatocytes are damaged [61, 62].

**Figure 4:**
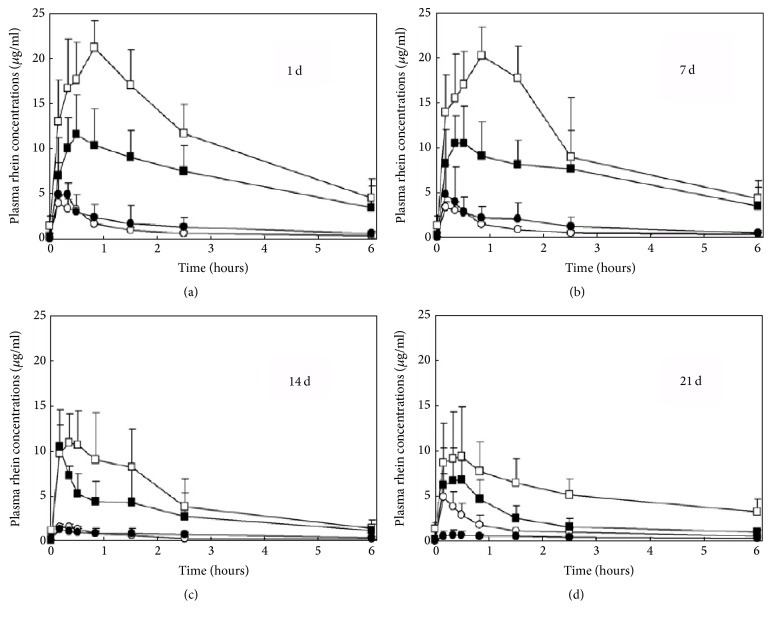
Profiles of average plasma concentration of rhein versus time after orally administrating* Rheum palmatum *extract. Normal rats and observed data of CRF are represented by opened and filled symbols. Low and high dosage groups are represented with circle and square, respectively [17].

Rhein, as the mainly absorbable anthraquinone derivative, is taken into systemic circulation after oral administration. Wang et al. carried out the toxicokinetic analysis of water extract of* Rheum palmatum* L. [17]. Their study suggested that rhein was majorly used to evaluate the toxicokinetics of rhubarb.  Figure 4 depicts the profiles of average concentration of rhein versus time. When given to the same dosage, the AUC, *C*_max *k*_, *t*_1/2_ *Ka,* and *t*_1/2_ of chronic renal failure (CRF) groups were generally lower than those of normal groups. As dose given increased, the AUC and *C*_max_ values did not increase proportionally in both chronic renal failure (CRF) rat model and normal groups. This might be on account of a nonlinear pharmacokinetic course, or because the indigestion led to severe diarrhea induced by the rhubarb itself at a high dosage. Therefore, the study suggests that the possibility of renal lesion using rhubarb in treatment of CRF would be limited when the dose was properly controlled.

### 5.2. Hepatic Transporter Study

AREHCs express hepatic transporter genes that are expressed by primary hepatocytes [60]. The mRNA expression level of the hepatic transporter genes is significantly higher compared to HepG2 cells. The expanded hepatocytes can be used for compound uptake and metabolism studies. The resazurin assay can be repetitively used to bioreactor cultures with pHH or HepG2 [63]. However, a toxic effect of resazurin upon long-term exposure was observed in other studies that could impair the diclofenac exposure effect.

### 5.3. Hepatotoxicity Technology

Primary hepatocytes and liver cell lines are important for in vitro toxicogenomic studies and RT-qPCR technology. Gene expression profiles following exposure to potential hepatotoxicants can be analyzed. Identification of reference gene with stable expression during in vitro toxicology studies is critical. A study performed by Fox et al. was aimed to analyze stabilities of reference genes in HepG2 and primary rat hepatocytes with two different culture systems [64]. The genes E2F7 and IL-11RA were then identified as potential toxicity biomarkers for acetaminophen treatment. Are hepatocytes can be used for hepatotoxicity studies and predictive screens of novel compounds? The expanded primary hepatocytes have high tolerance (IC_50_ > 200 *μ*M) to nontoxic compounds (such as phenytoin and ciprofloxacin). AREHCs are responsive to moderate hepatic toxins (such as methotrexate) and are highly sensitive (IC_50_ < 30 *μ*M) to hepatotoxins (such as ketoconazole and tacrolimus) [60].

### 5.4. Genotoxicity Technology

The validated hepatocytes can be used for micronucleus assays for genotoxicity application, when grown in AREHC Genotoxicity Assay Medium [60]. This medium permits hepatocyte cell division allowing the observation of micronucleus formation when genotoxic compounds were used to treat hepatocytes. Genotoxicity Assay Validated AREHCs can be used for micronucleus assays to determine potential genotoxic effects of novel compounds that alter cell division and DNA replication to be observed. Genotoxicity Assay Validated AREHCs are division-competent hepatocytes unlike uncultured primary hepatocytes and therefore have wider applicability and can be used for assays that require cell division such as micronucleus assays, for instance, the effect of cyclophosphamide on AREHC viability and micronucleus formation [60]. Cyclophosphamide is a DNA alkylating agent that interferes with DNA replication and is used for cancer chemotherapy. In a study by using a combination of metabolomic and genomic analyses, senecionine hepatotoxicity was determined. Significantly elevated conjugated bile acids after senecionine exposure were observed via serum profiling of bile acids. The hepatic mRNA levels of several key bile acid metabolism related genes were found to be changed significantly. Cholesterol 7-*α* hydroxylase, bile acid CoA-amino acid N-acetyltransferase, organic anion-transporting polypeptides, multidrug-resistance-associated protein, and sodium taurocholate cotransporting polypeptide were involved in the process. The cross-omics approach provides a comprehensive view for the study of the toxicity induced by senecionine, a hepatotoxic pyrrolizidine alkaloid. Moreover, the change in the respective transporters and bile acid metabolism could provide another toxic mechanism for pyrrolizidine alkaloids [65].

### 5.5. Culture Technology

A dynamic 3D bioreactor system cultured with primary human liver cells could be useful to investigate hepatic drug effects. It is suggested that a stable performance of the hepatocytes subsequent to three days of an adaption phase based on the data from untreated bioreactors (control BR), which is in the line with other studies that indicated hepatocyte functionality preserved in the 3D bioreactor, was longer than those in 2D cultures [66]. Therefore, it is a main advantage for the perfused 3D bioreactor system over conventional 2D cultivation to perform long-term in vitro toxicity studies [67]. A marked and rapid decrease of CYP gene expression has been observed when compared with the freshly isolated human hepatocytes [68]. In addition, Liver Sinusoidal Endothelial Cells are specialized scavenger cells that have a high capacity for uptake of soluble molecules up to 0.2 *μ*m in size. The liver endothelial cells can be cocultured with the expanded hepatocytes to determine paracrine signaling between these two cell types. Coculture can also be used to determine the effect of compounds or molecules on hepatocytes that are taken up exclusively by liver endothelial cells such as bile acids and phalloidin. Hepatic organoids can be grown by coculturing hepatocytes, Liver Sinusoidal Endothelial Cells, and Mesenchymal Stem Cells.

### 5.6. Bioinformatics in DILI/HILI Research

Currently, bioinformatics, including network pharmacology and network toxicology, is an important tool to study the effectiveness and safety of drugs. Network pharmacology has been developed from the principles of systems biology and network theory. Systems biology aimed to integrate all levels of biological organization including cell, organ, organism, or population to explain biological complexity. The concept of network pharmacology is established on the belief that, rather than individual molecules, targeting multiple nodes in interconnected molecular systems could result in enhanced efficacy and low occurrence of adverse effects [69, 70]. Combining systems biology with network biology may enable a new network pharmacology approach to be applied in drug research. The network of drug action is built by drug-target networks and biological networks. Thus, network pharmacology could be used to investigate the complex dynamics of interconnected molecular and organic systems in drug discovery, development, and drug safety [71, 72]. The recent progress in applying the network pharmacology in Chinese herbal medicine research is shown in the following seven aspects, such as predicting new drug targets, action mechanism, new drug discovery, drug evaluation for PD/PK, safety and toxicology, quality control, and bioinformatics.

Since Chinese herbal medicine is a very complicated system, the network toxicology, as an important branch of network pharmacology, in terms of safety prediction of CHM, faces three great challenges. It refers to study on the toxicological features and their interaction and regulation in biological systems and investigates the mechanism of toxicity. Network toxicology now develops rapidly in safety prediction of CHM. The application of network toxicology to safety and toxicology study on CHM is extremely beneficial to identify the toxic ingredients and potential incompatibility of CHM, including integration studies of bioinformatics, innovation of methods, and risk assessment in future development of the network toxicology in CHM research.

Network toxicology is another intriguing field for CHM research [73]. In network toxicology, adverse outcomes in human and toxicological mechanisms of medications can be investigated through network modeling of the complex relationships among adverse reactions, targets, and chemical entities [74]. Specifically, the knowledge about the chemical entities, genes, proteins, toxicological endpoints, and adverse reactions needed to be collected from literature, public database, and experiments. A network can be constructed based on comprehensive relationships among the nodes (such as genes, proteins, toxicological endpoints, and adverse reactions), and network analysis will help to infer the unknown relationships among the interesting nodes (such as active ingredients and targets). For the CHM studies, the network modeling can be utilized to identify the active toxic ingredients in herbs, to understand the toxicological mechanisms, and to predict the potential adverse outcomes in human and contradiction of herb combinations. Overall, network toxicology can be a promising tool that can provide the scientific evidences to support the safety evaluation of CHM.

**Figure 5:**
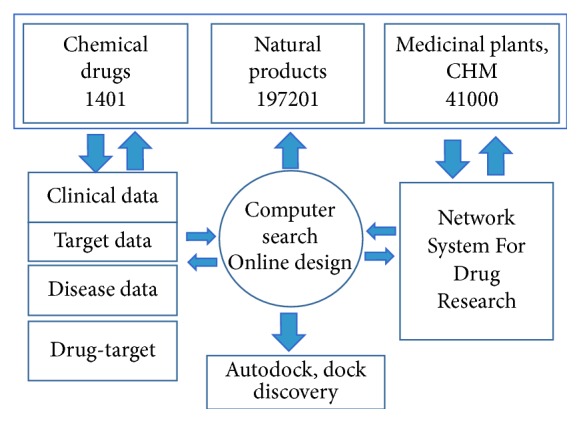
CHM Network pharmacology database.

Network toxicology of CHM aims at describing the network toxicology, which refers to the study on the toxicological properties of the model by building a network, using the established network model to analyze toxic substances interaction and regulation in biological systems, understanding the toxic effects of drugs or drug-drug on the body, and investigating the mechanisms of toxicity. CHM network pharmacology database is established by Guangdong Hospital of Traditional Chinese Medicine and Peking University for online use [75]. The basic framework of CHM Database is shown in Figure 5.

1401 chemical drugs, 197201 natural products, and 4100 herbal medicines or medicinal plants are collected. Tremendous amount of information obtained supports the following investigations: (1) CHM network is applied to predict the interaction of CHM molecules-pathway and formula contained; (2) interaction of target and target protein is used to study the mechanism of CHM; (3) the pharmacological responses of the active molecules from a population of molecules and combined drugs at the levels of multitargets, indications, side effects, and molecular-drug-targets-disease relationships are evaluated and summarized; (4) systems biology and translational medicine from basic research to clinical outcome are combined; and (5) the possible active molecules and active targets in CHM prescriptions are predicted [72]. The successful application of the network toxicology to safety and toxicology study of CHM is extremely beneficial to identify the toxic ingredients and potential incompatibility of CMMs. In the future, the network toxicology will develop rapidly in safety prediction of CHM by utilizing public and/or open database. The network toxicology may act as a new approach for screening the potential toxic ingredients in herbs.

## 6. Discussion and Conclusions

In China, more than 5000 species of recorded medicinal herbs are used in treatment of diseases. Over the past 15 years, a series of adverse events have taken place, in terms of safety, such as aristolochic acid in 2000,* Longdanxiegan* pill in 2003, and* Houttuynia* injection in 2006. Special consideration should be given to the safety issue of CHM, but effectiveness and efficacy of CHM cannot be less weighted.

Apart from China, Mexico owns the second largest number of herbs recorded with approximately 4500 species [76]. The use of medicinal plants by modern populations has been explored by many publications in Latin America and Mexico. In addition, most herb products are used for the purpose of health care in Japan and South Korea.

In Mexico, a total of 5% state flora is used for medicinal plants with 235 different herbs recorded for medicinal use. During the 1990s, ethnomedicinal study, combining ethnopharmacy and ethnobotany, was developed in Mexico and Latin America [77]. Based on the records in the library of traditional Mexican medicine, seven herbal products daily used in Mexico have been warned about due to hepatotoxicity:* Citrus aurantium* L. (citrus orange),* Tilia mexicana *Schlechtendal (tilia),* Scoparia dulcis* L. (maidenhair),* Prunus persica* L. (peach),* Morus alba* L. (white mulberry),* Equisetum hyemale* L. (horse tail), and* Rosmarinus officinalis* L. (rosemary). A prospective study between 2004 and 2013 to characterize hepatotoxicity and results, comparing herbal and dietary supplements (HDS) versus medications, was described by Navarro et al. [27]. Of the 839 subjects enrolled, 130 patients were sampled, the incidence of liver injury caused by bodybuilding and nonbodybuilding HDS was from 7% to 20%.

The clinical manifestations of HILI varied from asymptomatic or abnormal hepatic biochemical tests to acute liver failure [78]. 28 patients with DILI in the US DILI Network database were described by Chalasani et al. Hepatocellular injury was founded as the most common side effect in 63% of the patients sampled, and cholestatic injury was approximately 17%; of all DILI cases, 88% were mild to moderate and the rest were severe or fatal. Patients who required a liver transplant were limited and 9% of patients eventually developed chronic DILI [46].

A prospective nationwide study of DILI/HILI in South Korea performed by Suk et al. [79] reported 371 cases. The causes of DILI/HILI included herbal medications in 27.5% of cases, prescription or nonprescription medications in 27.3%, health foods or dietary supplements in 13.7%, medicinal herbs or plants in 9.4%, folk remedies in 8.6%, and herbal preparations in 3.2%.

According to the WHO Collaborating Centre for International Drug Monitoring reports, adverse reaction reports of herbs were 4960 cases prior to 1994. By the end of 1999, it has increased to 8986 cases; great concern has been given to safety issue of CHM. Since then, measures have been taken; for instance, the US Food and Drug Administration terminated use of the preparation almonds made; Singapore banned the import and sale of preparations containing berberine; Japan reevaluated the efficacy and safety of* Bupleurum* preparations; the use of aristolochic acid-containing medicines was prohibited, and sale of the Senecio plant medicines is banned in the UK.

As a matter of fact, it is hard to draw a conclusion that the safety issues of CHM become increasingly serious. The chemical composition of CHM is complex and diverse, but the content of toxic substances is minimal. It requires comprehensive investigation of adverse reactions and drug safety issues of CHM. Due to the complex of CHM, in terms of planting, processing, and other factors, it is not proper to make judgment on CHM by simply applying the standard and criteria to assess overall effect of CHM.

Based on the information available and literature, the authors analyzed and discussed the hepatotoxicity problem of CHM, including global overview on herbal-induced liver injury (HILI), current research progress on toxic CHM, diagnosis and treatment of HILI, and modern approaches and technologies of study of hepatotoxicity. A guideline for the diagnosis and treatment of HILI has now been drafted. The hepatotoxicity issue of Chinese herbal medicine, as a matter of fact, is overestimated. The investigation of hepatotoxicity of CHM is now booming with worldwide application of CHM. This review, therefore, provides useful information for investigating hepatotoxicity of herbal medicine and characterizing DILI caused by CHM. In addition, in which way further efforts should be made to study the rationale of CHM and liver injury has been discussed.

## Figures and Tables

**Table 1:** Selected herbal medicines listed in LiverTox.

Herbal medicines	Annotation in Chinese
*Polygonum multiflorum *Thunb.	*Heshouwu*
*Tripterygium wilfordii* Hook. f.	*Leigongteng *
*Bupleurum chinense* DC.	*Chaihu *
*Epimedium brevicornum* Maxim.	*Yinyanghuo*
*Silybum marianum* (L.) Gaertn.	*Shuifeiji*
*Cassia acutifolia *Delile	*Fanxieye *
*Rhamnus purshiana *DC. (cascara)	*Shuli*

**Table 2:** Typical toxic Chinese herbal medicines.

Source types	Chinese herbal medicines	Medicinal source
Plants	Radix Aconiti Kusnezoffii	*Aconitum kusnezoffii* Reichb.
	Radix *Aconiti penduli*	*Aconitum pendulum* N. Busch
	Rhizoma Arisaematis	*Arisaema erubescens* (Wall.) Schott
	Flos Daturae	Datura *metel *L.
	Rhizoma Typhonium	*Typhonium giganteum* Engl.
	Radix Aconiti Lateralis	*Aconitum carmichaelii* Debx.
	Rhizoma Pinellia	*Pinellia ternate* (Thunb.) Breit.
	Semen Nut-vomitive	*Strychnos nux-vomica* Linn.
	Herba Euphorbia	*Euphorbia kansui* L.
	Radix euphorbiae lantu	*Stellera chamaejasme* Linn.
	Garcinia	*Garcinia pedunculata *Roxb.
	Leptochloa	*Leptochloa chinensis* (L.) Ness
	Radix Rhododendroni molli	*Rhododendron molle* (Blume) G. Don
	Semen Hyoscyami	*Hyoscyamus niger* L.
	Croton	*Croton tiglium* L.

Animals	Venenum Bufonis	Asiatic toad
	Cantharidin	*Lytta caragana* Pallas
	Huechys sanguinea	*Huechys sanguinea* (De Geer)

Minerals	Arsenic	Arsenic trioxide
	Arsenolite	Arsenolite
	Arsenic stone	Arsenic stone
	Rabiagar	Arsenic disulfide
	Calomelas	Mercurous chloride
	Hydrargyri oxydum Rubrum	Hydrargyri oxydum Rubrum
	Mercury	Mercury

**Table 3:** Chinese medicines with HILI on animals.

Chinese herbal medicines	Annotation in Chinese	Medicinal plant
Anisi Stellati Fructus	*Bajiao*	*Illicium verum* Hook. f.
Radix Sanguisorbae	*Diyu*	*Sanguisorba officinalis* L.
Fructus *Gardeniae*	*Zhizi*	*Gardenia jasminoides* Ellis
Galla Chinensis	*Wubeizi*	*Rhus chinensis* Mill
Cortex Granati	*Shiliupi*	*Punica granatum* L.
Chebulae Fructus	*Hezi*	*Terminalia chebula *Retz.
Rhizoma Acori tatarinowii	*Shichangpu*	*Acorus tatarinowii* Schott
Fructus Foeniculi	*Xiaohuixiang*	*Foeniculum vulgare* Mill.
Cortex Cinnamomi	*Guipi*	*Cinnamomum cassia* Presl
Fructus Aristolochiae	*Madouling*	*Aristolochia debilis* Sieb. et Zucc.
Caulis Akebiae	*Mutong*	*Akebia quinata* (Thunb.) Decne

**Table 4:** Herbal medicines with HILI used in treatment.

Chinese herbal medicines	Annotation in Chinese	Medicinal plant
Cortex Albiziae	*Hehuanpi*	*Albizia julibrissin *Durazz
Semen Strychni	*Maqianzi*	*Brucea javanica* (L.) Merr.
Rhizoma Alismatis	Zexie	*Alisma orientale *(Sam.) Juzep.
Folium Sennae	*Fanxieye*	*Cassia angustifolia* Vahl.
Semen *Cassiae *	*Juemingzi*	*Cassia tora* Linn.
Herba Chenopodii	*Tujinjie*	*Chenopodium ambrosioides *L.
Folium *Clerodendri*	*Chouwutong*	Clerodendrum trichotomum Thunb.
Radix Dioscoreae bulbifera	*Huangdu*	*Dioscorea bulbifera* Linn
Omoto Nipponlily	*Wannianqing*	*Rohdea japonica *(Thunb.) Roth
Herba Ephedrae	*Mahuang*	*Ephedra sinica* Stapf
Herba Gynurae	*Jusanqi*	Gynura japonica (Thunb.) Juel.
Semen Hydnocarpi Hainanensis	*Dafengzi*	Hydnocarpi Hainanensis (Merr.) Sleum.
Folium Ilexi pubescensi	*Maodongqing*	*Ilex pubescens *Hook. et Arn.
Fructus Trichosanthis	*Gualou*	*Trichosanthes kirilowii* Maxim.
Fructus Meliaceae	*Kulianzi*	*Melia azedarach *L.
Semen *Myristicae*	*Roudoukou*	*Myristica fragrans* Houtt.
Herba Papaveri somniferi	*Yingsu*	*Papaver somniferum* L.
Radix Phytolaccae	*Shanglu*	*Phytolacca acinosa* Roxb.
Radix Polygoni cuspidati	*Huzhang*	*Polygonum cuspidatum* Sieb. et Zucc.
Radix Polygoni multiflori	*Heshouwu*	*Polygonum multiflorum *Thunb.
Rhubarb	*Dahuang*	*Rheum officinale* Baill.
Semen Ricini	*Bimazi*	*Ricinus communis *L.
Radix et Rhizoma Salviae miltiorrhizae	*Danshen*	*Salvia miltiorrhiza *Bunge
Radix Scutellariae	*Huangqin*	*Scutellaria baicalensis* Georgi
Herba Senecioe scandensi	*Qianniguang*	*Senecio scandens* Buch.-Ham. ex D. Don
Herba Scutellariae Barbatae	*Banzhilian*	*Scutellariae Barbatae* D. Don
Radix Stephaniae tetrandrae	*Hanfangji*	*Stephania tetrandra* S. Moore
Flos Syzygii aromatici	*Dingxiang*	*Syzygium aromaticum* (L.) Merr. et Perry
Herba Taxilli	*Sangjisheng*	*Taxillus sutchuenensis* (Lecomte) Danser
Radix et Rhizoma Tripterygii	*Leigongteng*	*Tripterygium wilfordii* Hook. f.
Herba Typhae	*Xiangpu*	*Typha angustifolia* L.
Radix Valerian	*Xiecao*	*Valeriana officinalis* L.
Fructus Xanthii	*Cangerzi*	*Xanthium sibiricum *Patrin ex Widder

**Table 5:** CHM formulations with DILI.

Formulations	CHM preparations
Pill	*Zhuangguguanjie* pill; *Baishi* pill; *Liushen* pill; *Shiduqing* pill; *Xuedu* pill; *Zhifeng-tougu* pill; *Xiaokechuan* pill; *Tianma* pill
Decoction	*Xiaochaihu* decoction; *Dachaihu* decoction; *Gegen* decoction; *Dahuangmudanpi* decoction
Capsule	Complex *Qingdai *capsule; *Baidianfeng* capsule;* Zhuangushenjin* capsule; *Diaoxinxuekang* capsule
Tablet	*Xiaoying *tablets; *Xiaohe* tablets;* Guxian* tablets; *Zengshengping* tablets; *Niuhuang Jiedu *tablets;* Kunming-Shanhaitang *tablets
Granule	*Baifukang* granule
Injection	Complex *Danshen* injection
Powder	*Ganji* powder;* Fangfeng-Tongsheng* powder

**Table 6:** Categories of chemical substance with HILI.

Categories	Substance with HILI
Alkaloids	Aconitine; febrifugine; ajmaline; vincristine
Glycosides	Cardiac glycosides; cyanogenic glycosides; saponins; *bulbifera*
Toxic proteins	toxin fruit; croton, castor; abrinjatropha; trichosanthes; centipede; snake venom; viper
Terpene and lactones	Toosendan; spurge; *Coriaria* leaf; wormwood leaves
Tannins	Gall; peel; holly leaf
Heavy metals	Cinnabar; realgar; light powder; litharge; vitriol; red lead
Other toxic components	*Garcinia*; red fennel root; *Hydnocarpus*, *Pinellia ternata*; daphne; melon pedicle acids; *Asarum*; rue; mint; *Asarum forbesii*; musk grass
